# Conditions identified by common comprehensive geriatric assessment measures and subsequent acute care among adults with SLE

**DOI:** 10.1136/lupus-2025-001935

**Published:** 2026-04-08

**Authors:** Laura C Plantinga, Jessica Fitzpatrick, Charmayne M Dunlop-Thomas, Courtney Hoge, S Sam Lim, Jinoos Yazdany, Patricia P Katz, C Barrett Bowling

**Affiliations:** 1 Department of Medicine, University of California San Francisco, San Francisco, California, USA; 2 Department of Medicine, Emory University, Atlanta, Georgia, USA; 3 Department of Behavioral, Social, and Health Education Sciences, Emory University, Atlanta, Georgia, USA; 4 Department of Medicine, Duke University, Durham, North Carolina, USA

**Keywords:** Epidemiology, Lupus Erythematosus, Systemic, Outcome Assessment, Health Care

## Abstract

**Objective:**

The value of comprehensive geriatric assessment (CGA) in the setting of chronic disease, regardless of age, has been increasingly recognised. We examined whether geriatric conditions identified by measures commonly used in CGA were associated with subsequent acute care utilisation among individuals with SLE.

**Methods:**

In this longitudinal cohort study, data from participants from a population-based cohort of adults (≥18 years) with validated SLE were included if they completed a study visit during which data on CGA measures were collected and a subsequent (12–24 months later) questionnaire that included items on emergency department (ED) visit and hospital admissions. Associations (incidence rate ratios (IRRs)) of CGA-identified conditions (impairments in physical or cognitive performance; limitations in self-reported physical function or instrumental or basic activities of daily living (ADLs); restrictions in community mobility; polypharmacy; urinary incontinence) with ED visits and hospital admissions were assessed with multivariable negative binomial models, adjusting for demographic and clinical characteristics.

**Results:**

Nearly all (97.1%) participants (n=241; mean age, 45.9; 93.0% female; 83.8% black) had at least one CGA-identified condition. Those with CGA-identified conditions with physical performance (IRR=1.69, 95% CI 1.17 to 2.43), cognitive performance (IRR=2.24; 95% CI 1.32 to 3.79), self-reported physical function (IRR=1.77; 95% CI 1.10 to 2.85), basic ADLs (IRR=1.55, 95% CI 1.09 to 2.20) and falls (IRR=1.57; 95% CI 1.08 to 2.27) had higher rates of ED visits. However, after full adjustment, only cognitive impairment was statistically significantly associated with ED visits (IRR=1.79; 95% CI 1.05 to 3.03) and hospital admissions (IRR=2.44; 95% CI 1.20 to 4.96).

**Conclusions:**

Identification of CGA-identified conditions, particularly cognitive impairment, may be useful for mitigating the risk of subsequent acute care utilisation among patients with SLE. Future studies should examine the effectiveness of CGA measures in improving other important outcomes, such as patient quality of life and satisfaction.

**WHAT IS ALREADY KNOWN ON THIS TOPIC**

**WHAT THIS STUDY ADDS**

- While we found that assessment of measures commonly used in CGA identified at least one condition, such as impaired function, activities of daily living and falls, in most (97%) participants in an adult population-based cohort, only impaired cognitive function was associated with subsequent acute care utilisation.

**HOW THIS STUDY MIGHT AFFECT RESEARCH, PRACTICE OR POLICY**

- Future studies should examine the effectiveness of incorporating CGA measures and, especially, geriatrician-led CGA in SLE care, not only in preventing acute care but also in improving other important outcomes, such as patient quality of life and satisfaction with care.

## Introduction

 Comprehensive geriatric assessment (CGA) is a multidisciplinary, multidimensional method designed to manage the health of older adults and includes assessments of physical medical condition, mental health, function, activities of daily living (ADLs), patient-prioritised life roles, social circumstances and environment.^1^ While primarily associated with geriatric care, the value of CGA has been increasingly recognised for chronic diseases, including not only those associated with older age (eg*,* cancer,^2^,^4^ chronic kidney disease)^5^,^7^ but also those that often occur among younger populations (eg*,* HIV,^8^ multiple sclerosis).^9^ Across these varied settings, CGA has been shown to be associated with better patient and provider perceptions of the usefulness and quality of delivered care,^2 7^ better patient quality of life^3^ and increased patient-centredness of treatment decisions and goal-setting.^4^,^6^

Similar to these conditions, SLE involves chronic, widespread inflammation and accumulation of morbidity, which may parallel the inflammation^10^ and multimorbidity of ageing^11^; in fact, there is evidence of accelerated ageing in SLE.^12^ Those with SLE are simultaneously experiencing chronological ageing, with advances in treatments,^13^ overall ageing of the population^14^ and late diagnoses (one in five after age 60).^15 16^ Together, these patterns suggest that CGA may be valuable in the setting of SLE.

The avoidance of acute care utilisation is one metric by which the value of CGA can be assessed. We previously performed several assessments that are commonly measured components of a CGA (table 1) as part of an ancillary study. Here, by linking these data to follow-up acute utilisation data reported subsequently in the ongoing parent cohort study, we estimate associations between individual CGA-identified conditions—as well as the total number of conditions—with subsequent emergency department (ED) visits and hospital admissions.

**Table 1:** Primary and alternate operationalisations of conditions identified by CGA

CGA-identified condition	Measure (description)	Minutes to administer	Range	Primary categorisation	Alternate (more restrictive) categorisation	As continuous score
Physical performance	SPPB (balance, chair stands, 4 m gait speed)	∼10	0–12 (higher=better)	SPPB score <10	SPPB score ≤6	Per+1-point difference in SPPB score
Cognitive performance	TMTB (connecting numbers and letters in alternating sequence)	∼5	0–300 s (test stopped at 300 s)	TMTB time >1.5 SD greater than normative value for age and education	TMTB time >2 SD greater than normative value for age and education	Per+1 min difference in time to complete TMTB
	CLOX (clock drawing)	∼5	0–15 for each (higher=better)	CLOX1 ≤10 or CLOX2 ≤12	CLOX1+CLOX2 <28 (<75th percentile)	Per+1-point difference in CLOX1+CLOX2
	Overall (combined)	∼10	—	Either impaired in TMTB or CLOX	Impaired in both TMTB and CLOX	—
Self-reported physical functioning	PROMIS Physical Functioning 12a (12-item assessment of physical abilities)	∼2	13.3–66.1 (higher=better; mean=50, SD=10)	T-score <35 (>1.5 SD below population norm)	T-score <30 (>2 SD below population norm)	Per+10-point difference (1 SD) in T-score
Activities of daily living	IADLs (eg*,* preparing food, household chores, managing medications/finances)	∼2	6–18 (score; higher=better)	At least some difficulty in at least one IADL	At least moderate difficulty in at least one IADL	Per+1-point difference in IADL score
	BADLs (eg*,* bathing, transferring, dressing)	∼2	8–24 (score; higher=better)	At least some difficulty in at least one BADL	At least moderate difficulty in at least one BADL	Per+1-point difference in BADL score
Falls	Falls, FES (self-reported falls and fear of falling)	~3	Falls, any integer FES, 0–100 (higher=worse)	At least one fall in the prior year	At least two falls in the prior year or FES score >70	Per+1 fall in prior year
Community mobility	UAB-LSA; (frequency and help required to reach life-space levels)	~2	0–120 (higher=better)	UAB-LSA score <60	Not making it to neighbourhood level at least weekly without any help from a device or person	Per+10-point difference in UAB-LSA score
Polypharmacy	Self-reported prescription and OTC medications	~2	Number of medications, any integer	≥5 medications	≥10 medications	Per+1 medication
Urinary incontinence	NHANES questionnaire items (urine leakage, frequency and amount), ISI	~2	ISI (amount × frequency), 0–12 (higher=more severe)	Leakage at least monthly	Leakage at least weekly	Per+1-point increase in ISI

BADL, basic activity of daily living; CGA, comprehensive geriatric assessment; CLOX, clock draw; FES, Falls Efficacy Scale; IADL, instrumental activity of daily living; ISI, Incontinence Severity Index; NHANES, National Health and Nutrition Examination Survey; OTC, over-the-counter; PROMIS, Patient-Reported Outcomes Measurement Information System; SPPB, Short Physical Performance Battery; TMTB, Trail Making Test B; UAB-LSA, University of Alabama Life-Space Assessment.

## Methods

### Study population and data sources

Approaches to Positive, Patient-centred Experience of Ageing with Lupus (APPEAL) was an ancillary study designed to understand the epidemiology of geriatric syndromes among adults with SLE. APPEAL participants were recruited for a single study visit (October 2019–May 2022) from the Georgians Organized Against Lupus (GOAL) cohort. GOAL is an ongoing, population-based SLE cohort in metropolitan Atlanta that aims to better understand disparities in SLE access to care, utilisation and outcomes.^17^ GOAL participants are adults (≥18 years) with a documented SLE diagnosis (≥4 revised American College of Rheumatology (ACR) criteria,^18^ or 3 ACR criteria plus a board-certified rheumatologist’s diagnosis). Additional inclusion criteria for APPEAL were: active participation in GOAL, ability to speak English, sufficient vision and hearing to see study materials and hear study instructions, ability to consent and living in Georgia at recruitment.

Baseline data for this cohort study were obtained from a series of performance tests and questionnaires administered by trained research coordinators during APPEAL visits, as well as data from the GOAL survey closest to the APPEAL visit date. While APPEAL was not designed for the administration of a CGA, many of the measurements selected for APPEAL are common components of a geriatrician-led CGA; we considered these our baseline exposures (table 1). Follow-up health utilisation data were obtained from the first GOAL survey that was ≥12 months after the APPEAL visit (to include the entire 12-month look-back period for health utilisation outcomes) and ≤24 months after the APPEAL visit (online supplemental efigure 1). Among 451 participants who completed APPEAL visits, those who had potentially invalid survey response patterns (n=4), did not have estimates of all selected CGA components (n=57) or did not complete a GOAL survey within 12–24 months of their APPEAL visit (n=149) were excluded, leaving n=241 (online supplemental efigure 2).

### Variables

#### Acute care utilisation

Participant-reported numbers of ED and hospital visits were assessed with the items ‘How many times have you visited the ER in the last 12 months?’ and ‘How many times were you admitted to the hospital in the last 12 months?’ during annual GOAL surveys.

#### CGA-identified conditions

Measures that were assessed at the APPEAL visit and that are common components of CGAs were included.

*Physical performance:* Physical performance was assessed using the Short Physical Performance Battery (SPPB).^19^ Impairment^20 21^ was defined as in table 1.

*Cognitive performance:* Cognitive performance was assessed with the Trail Making Test B (TMTB)^22^ and the clock draw (CLOX),^23^ two short assessments that can be administered quickly in a clinical setting.^24^ Impairments in the TMTB^25^ and CLOX,^23 26^ separately and combined, were defined as in table 1.

*Self-reported physical functioning:* The Patient-Reported Outcomes Measurement Information System (PROMIS) Physical Functioning-Short Form 12a^27^ was used to assess self-reported limitations in physical tasks. Limited physical functioning was defined as in table 1.

*ADLs*: The ability to perform ADLs, which can reflect an individual’s physical, cognitive and emotional functioning, as well as their available environment and resources, was assessed with the instruments from Lawton and Brody (instrumental ADLs (IADLs), such as shopping and housekeeping)^28^ and Katz *et al* (basic ADLs (BADLs), such as bathing and dressing).^29^ ADL limitations were defined as in table 1.

*Falls:* Participants were asked at the APPEAL visit if they had fallen in the past year and, if so, how many times,^30^ as well as about their fear of falling in daily situations (Falls Efficacy Scale).^31^ Falls were defined as in table 1.

*Restrictions in community mobility:* The University of Alabama Birmingham Life-Space Assessment (UAB-LSA)^32^ was used to measure community mobility. Restrictions in community mobility^32 33^ were defined as in table 1.

*Polypharmacy:* We defined polypharmacy as ≥5 prescribed or over-the-counter medications reported by the participant, versus 0-4,^34 35^ excluding eye drops, topical agents, inhalants and supplements (table 1).

*Urinary incontinence:* Urinary incontinence data were collected via a questionnaire adapted from the National Health and Nutrition Examination Survey.^36^ Urinary incontinence was defined as in table 1.

#### Other variables

Other variables of interest were informed by our conceptual model (online supplemental efigure 3). Age, sex (at birth), race (black, white, other), ethnicity and education (high school graduate/equivalency or lower, some college/associates degree and college graduate or higher) were self-reported during the APPEAL visit. Insurance status (categorised as none, Medicaid, Medicare and private) was assessed from the closest GOAL survey. Body mass index (BMI) was calculated from measured or reported weight and height; obesity was defined as BMI ≥30 kg/m^2^. Disease duration was derived from the date of diagnosis self-reported on the closest GOAL survey. Current SLE activity was assessed during the APPEAL visit via the Systemic Lupus Activity Questionnaire (SLAQ; range 0–44; higher scores=greater SLE-related disease activity).^37^ The Brief Index of Lupus Damage (BILD) score (range, 0–46; higher scores=greater cumulative SLE-related organ damage)^38^ closest to the APPEAL visit was obtained from GOAL. The use of steroids and the use of any immunosuppressants (any of: methotrexate, cyclophosphamide, cyclosporine, mycophenolate, dapsone, azathioprine, belimumab, rituximab or tumour necrosis factor inhibitors) were self-reported during the APPEAL visit. Depressive symptoms were assessed via the validated 8-item PROMIS Depression Short Form-8a^39^ and reported as T-scores (where 50=mean score and 10=1 SD). The gap between the APPEAL visit and the start of the look-back follow-up period was calculated as the difference between the date of the subsequent GOAL visit and the date of the APPEAL visit, subtracting 12 months (online supplemental efigure 1).

### Statistical analysis

Participant characteristics were described using summary statistics. For both ED visits and hospital admissions, the percentages with at least one event and the rate of the events per person-year were calculated, stratified by CGA components. Associations of each CGA-identified condition and the total number of CGA-identified conditions with ED visits and hospital admissions were estimated with incidence rate ratios from unadjusted and adjusted negative binomial models, accounting for potential overdispersion and zero inflation. Potential confounders were identified a priori (online supplemental efigure 3) and included if they were not considered mediators or were not accounted for by adjustment for other variables on the path. Sensitivity analyses included: (1) comparison of characteristics among those included versus excluded from our analysis, to address potential selection bias; (2) sequential adjustment to show models without SLAQ, BILD and depressive symptoms, as well as additional adjustment for steroids and immunosuppressants and for the gap between the APPEAL visit and start of look-back follow-up, to address possible misspecification of our conceptual model of confounding and mediation; (3) examining associations with all available data for each CGA component; (4) restricting models to those with a maximum of 6 months between the APPEAL visit and start of look-back follow-up (maximum of 18 months), to address the potential of bias due to missed events in this gap; and (5) examining associations with more restrictive categorisations of geriatric syndromes and with continuous scores (table 1), to address potential misclassification due to cut-off selection. Complete case analysis was used. The statistical significance threshold was set at 0.05. All analyses were conducted using Stata V.19.5 (College Station, Texas, USA).

### Patient and public involvement

Patients were involved in the design and conduct of this research. We used feedback from our pilot study of patient participants to create our initial protocol; participant feedback was also used to modify the protocol as needed throughout the course of this study. Participant burden was carefully considered in the number and order of measures assessed; participants were able to skip assessments and take breaks as needed. Finally, GOAL participants are informed of study results through regular study newsletters, which are suitable for a non-specialist audience.

## Results

### Participant characteristics

Mean age was 45.9 years, with 41.5% of participants being ≥50 years old (table 2). Most were female (93.0%), black (83.8%) and non-Hispanic (94.2%). Other demographic and clinical characteristics are shown in table 2. The median gap between the APPEAL geriatric assessment and the start of follow-up for acute care utilisation was 8.2 months. There were no statistically significant differences in demographic or clinical characteristics between included and excluded participants (online supplemental etable 1).

**Table 2:** Characteristics of included participants at the time of geriatric assessment

Characteristic*	Value
Demographic	
Age in years, mean (SD)	45.9 (11.7)
Age, *n* (%)	
18–34 years	53 (22.0)
35–49 years	88 (36.5)
≥50 years	100 (41.5)
Sex assigned at birth, *n* (%)	
Female	224 (93.0)
Male	17 (7.0)
Race, *n* (%)	
Black	202 (83.8)
White	24 (10.0)
Other	15 (6.2)
Ethnicity, *n* (%)	
Non-Hispanic	226 (94.2)
Hispanic	14 (5.8)
Educational attainment, *n* (%)	
High school degree or less	56 (23.2)
Some college/associates degree	90 (37.3)
College graduate or higher	95 (39.4)
Insurance, *n* (%)	
None	28 (12.1)
Medicaid	44 (19.0)
Medicare	84 (36.2)
Private	76 (32.8)
Clinical	
Disease duration in years, median (IQR)	14.7 (9.2–22.7)
SLAQ score, median (IQR)	11 (7–15)
BILD score, median (IQR)†	2 (1–4)
Obese, *n* (%)	
Yes	106 (45.3)
No	128 (54.7)
Currently taking steroid, *n* (%)	
Yes	98 (40.8)
No	142 (59.2)
Currently taking immunosuppressant, *n* (%)	
Yes	127 (52.9)
No	113 (47.1)
Depressive symptoms T-score, mean (SD)‡	48.5 (8.9)
Time between geriatric syndrome assessment and start of follow-up for acute care utilisation in months, median (IQR)	8.2 (4.1–10.4)
CGA-identified conditions	
Physical performance, *n* (%)	
Impaired	136 (56.4)
Not impaired	105 (43.6)
Cognitive performance, *n* (%)	
TMTB or CLOX	
Impaired	196 (83.3)
Not impaired	45 (16.7)
TMTB only	
Impaired	153 (63.5)
Not impaired	88 (36.5)
CLOX	
Impaired	132 (54.8)
Not impaired	109 (45.2)
Self-reported physical function, *n* (%)	
Limited	32 (13.3)
Not limited	209 (86.7)
IADLs, *n* (%)	
Limited	135 (56.0)
Not limited	106 (44.0)
BADLs, *n* (%)	
Limited	101 (41.9)
Not limited	140 (58.1)
Falls, *n* (%)	
Yes	71 (29.5)
No	170 (70.5)
Community mobility, *n* (%)	
Restricted	116 (48.1)
Not restricted	125 (51.9)
Polypharmacy, *n* (%)	
Yes	131 (54.4)
No	110 (45.6)
Urinary incontinence, *n* (%)	
Yes	78 (32.4)
No	163 (67.6)
Total no. of geriatric conditions, *n* (%)	
0–1	29 (12.0)
2–3	83 (34.4)
4–5	70 (29.1)
6–8	59 (24.5)

IQR=(25th–75th percentile).

* *N*=241 for all except: ethnicity (n=240), insurance status (n=232), disease duration (n=240), SLAQ (n=232), obesity (n=234), medications (n=240) and depressive symptoms (n=237).

† Measured from the closest Georgians Organized Against Lupus (parent study) assessment.

‡ From the PROMIS Depression Short Form-8a.

BADLs, basic activities of daily living; BILD, Brief Index of Lupus Damage (range, 0–46; 46 is maximum damage); CGA, comprehensive geriatric assessment; CLOX, clock draw; IADLs, instrumental activities of daily living; PROMIS, Patient-Reported Outcomes Measurement Information System; SLAQ, Systemic Lupus Activity Questionnaire (range, 0–47; 47 is maximum activity); TMTB, Trail Making Test B.

The percentage with individual CGA-identified conditions ranged from 13.3% (self-reported physical function) to 83.3% (TMTB or CLOX impairment). Nearly all (97.1%) participants had at least one CGA-identified condition (online supplemental efigure 4) using our primary definitions, with 12.0%, 34.4%, 29.1% and 24.5% having 0–1, 2–3, 4–5 and 6–8 CGA-identified conditions (table 1).

### Associations between conditions identified by comprehensive geriatric assessment and emergency department visits

#### Physical performance

Impaired physical performance was associated with 69% higher rates of subsequent ED visits, although adjustment for clinical factors attenuated this association (table 3). Results were unchanged with adjustment for the gap and for steroid and immunosuppressant use and similar when the gap was limited to 6 months, when all available physical performance data were used and when the more restrictive definition was used; each additional point on the SPPB was associated with a 9% lower rate of subsequent ED visits (online supplemental etable 2).

**Table 3:** Association of conditions identified by CGA with subsequent self-reported emergency department visits

CGA-identified condition (primary definition)	*n*/*N* (%) with any ED visit	Incidence of ED visits (ppy)	Incidence rate ratio (95% CI)*		
			Unadjusted	Adjusted for demographics	Adjusted for demographics and clinical factors
Physical performance					
Impaired	74/136 (54.4)	1.30	1.69 (1.17 to 2.43)	1.58 (1.09 to 2.29)	1.21 (0.83 to 1.76)
Not impaired	48/105 (45.7)	0.77	1.00 (Ref.)	1.00 (Ref.)	1.00 (Ref.)
Cognitive performance					
TMTB or CLOX					
Impaired	105/196 (53.6)	1.19	2.24 (1.32 to 3.79)	2.07 (1.20 to 3.56)	1.79 (1.05 to 3.03)
Not impaired	17/45 (37.8)	0.53	1.00 (Ref.)	1.00 (Ref.)	1.00 (Ref.)
TMTB only					
Impaired	81/153 (52.9)	1.24	1.61 (1.10 to 2.35)	1.54 (1.03 to 2.27)	1.36 (0.92 to 2.00)
Not impaired	41/88 (46.6)	0.77	1.00 (Ref.)	1.00 (Ref.)	1.00 (Ref.)
CLOX					
Impaired	74/132 (56.1)	1.20	1.30 (0.91 to 1.87)	1.22 (0.85 to 1.75)	1.26 (0.89 to 1.79)
Not impaired	48/109 (44.1)	0.92	1.00 (Ref.)	1.00 (Ref.)	1.00 (Ref.)
Self-reported physical function					
Limited	20/31 (62.5)	1.72	1.77 (1.10 to 2.85)	1.72 (1.07 to 2.77)	1.24 (0.73 to 2.11)
Not limited	102/209 (48.8)	0.97	1.00 (Ref.)	1.00 (Ref.)	1.00 (Ref.)
IADLs					
Limited	71/135 (52.6)	1.23	1.42 (0.99 to 2.03)	1.31 (0.91 to 1.90)	0.97 (0.65 to 1.45)
Not limited	51/106 (48.1)	0.87	1.00 (Ref.)	1.00 (Ref.)	1.00 (Ref.)
BADLs					
Limited	58/101 (57.4)	1.35	1.55 (1.09 to 2.20)	1.34 (0.93 to 1.93)	0.98 (0.66 to 1.45)
Not limited	64/140 (45.7)	0.87	1.00 (Ref.)	1.00 (Ref.)	1.00 (Ref.)
Falls					
Yes	40/71 (56.3)	1.44	1.57 (1.08 to 2.27)	1.43 (0.99 to 2.09)	1.29 (0.88 to 1.88)
No	82/170 (48.2)	0.92	1.00 (Ref.)	1.00 (Ref.)	1.00 (Ref.)
Community mobility					
Restricted	60/116 (51.7)	1.09	1.04 (0.73 to 1.49)	1.06 (0.74 to 1.53)	0.73 (0.50 to 1.05)
Not restricted	62/125 (49.6)	1.05	1.00 (Ref.)	1.00 (Ref.)	1.00 (Ref.)
Polypharmacy					
Yes	72/131 (55.0)	1.18	1.24 (0.87 to 1.78)	1.38 (0.95 to 2.01)	1.09 (0.75 to 1.58)
No	50/110 (45.5)	0.95	1.00 (Ref.)	1.00 (Ref.)	1.00 (Ref.)
Urinary incontinence					
Yes	45/78 (57.7)	1.15	1.12 (0.77 to 1.63)	1.15 (0.78 to 1.68)	0.90 (0.61 to 1.33)
No	77/163 (47.2)	1.03	1.00 (Ref.)	1.00 (Ref.)	1.00 (Ref.)

Demographics: age, sex, race and insurance; clinical: depressive symptoms, Systemic Lupus Activity Questionnaire score and Brief Index of Lupus Damage score.

* From negative binomial regression.

BADL, basic activity of daily living; CGA, comprehensive geriatric assessment; CLOX, clock draw; ED, emergency department; IADL, instrumental activity of daily living; ppy, per person-year; TMTB, Trail Making Test B.

#### Cognitive performance

Impaired cognitive performance was associated with 2.2-fold higher rates of subsequent ED visits; after adjustment for clinical factors, impaired cognitive performance was associated with 79% higher rates of subsequent ED visits (table 3). Results were unchanged with adjustment for the gap and for steroid and immunosuppressant use and similar when the gap was limited to 6 months, when all available TMTB and CLOX data were used, and when the more restrictive definition of cognitive performance impairment was used (online supplemental etable 2). Impaired TMTB performance was associated with 60% higher rates of ED visits, but results were not statistically significant after adjustment for clinical factors; impaired CLOX performance was not statistically significantly associated with ED visits (table 3). Results for TMTB and CLOX impairment were unchanged with adjustment for the gap and similar, but not statistically significant, when the gap was limited to 6 months. When the more restrictive definitions were used, CLOX performance impairment was associated with 70% higher rates of ED visits. Each additional minute taken on the TMTB was associated with a 24% higher rate of subsequent ED visits, but the association was not statistically significant (online supplemental etable 2).

#### Self-reported physical function

Limited self-reported physical function was associated with 77% higher rates of subsequent ED visits; this association was no longer statistically significant after adjustment for clinical factors (table 3). Results were similar in sensitivity analyses, although the association was 2.4-fold when all available self-reported physical functioning data were used and not statistically significant when the more restrictive definition was used (online supplemental etable 2).

#### Activities of daily living

IADL and BADL limitations were associated with 42% and 55% higher rates of subsequent ED visits, respectively, but only the association of BADL limitations with ED visits was statistically significant. Further, adjustment for clinical factors rendered both associations null (table 3). Adjustment for time between APPEAL and for steroid and immunosuppressant use and the start of follow-up for ED visits did not change the results, nor did restriction of the gap to 6 months or use of all available ADL data. The more restrictive definition of BADLs was associated with a twofold higher rate of ED visits, and each additional limitation in IADLs and BADLs was associated with 8% and 7%, respectively, higher rates of ED visits (online supplemental etable 2).

#### Falls

Self-reported falls in the prior year were associated with a 57% higher rate of subsequent ED visits; after adjustment for clinical factors, the association was not statistically significant (table 3). Adjustment for time between APPEAL and start of the follow-up period for ED visits, adjustment for steroid and immunosuppressant use and using all available falls data did not change the results, but restricting the gap to 6 months resulted in a non-statistically significant association. The more restrictive definition was associated with an 87% higher rate of ED visits, and each additional fall was associated with a 16% higher rate of ED visits (online supplemental etable 2).

#### Community mobility

Neither restricted community mobility, regardless of adjustment, nor the continuous UAB-LSA score was associated with ED visits (table 3; online supplemental etable 2). Results were similar with adjustment for steroid and immunosuppressant use and using all available community mobility data. The more restrictive definition was associated with a 40% higher rate of ED visits, but the association was not statistically significant (online supplemental etable 2).

#### Polypharmacy

Polypharmacy was not statistically significantly associated with subsequent ED visits, regardless of adjustment (table 3). Sensitivity analyses showed similar results, except that, in analyses in which the time between polypharmacy assessment and start of follow-up was restricted to 6 months, polypharmacy was associated with 85% higher rates of ED visits; further, each additional medication was associated with a 16% higher rate of ED visits (online supplemental etable 2).

#### Urinary incontinence

Urinary incontinence was not associated with subsequent ED visits, regardless of adjustment (table 3). Each point higher in the incontinence severity index was associated with a 5% higher rate of ED visits, but this association was not statistically significant (online supplemental etable 2).

#### Total number of CGA-identified conditions

**Figure 1:**
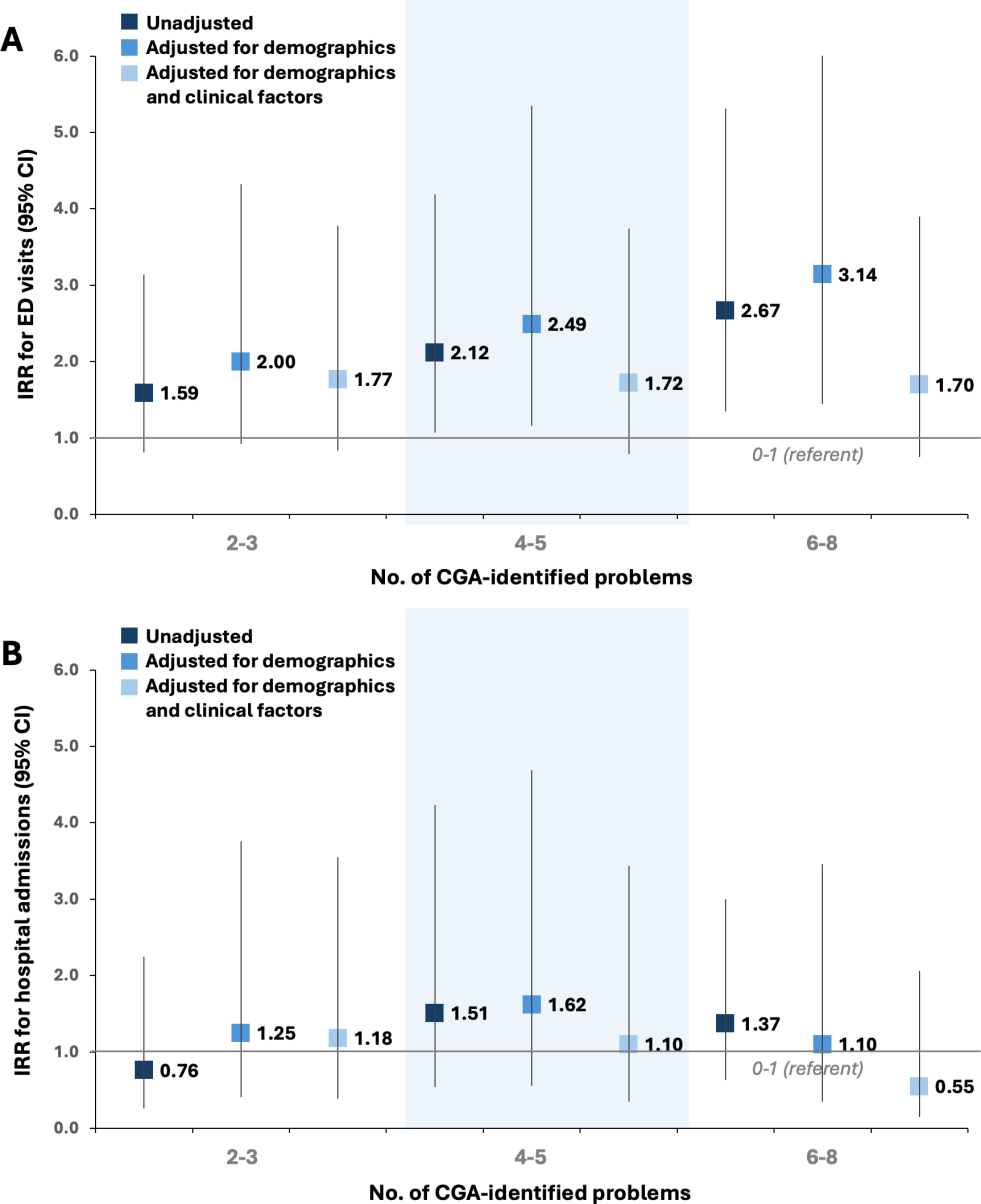
Association of the total number of conditions identified by CGA components with subsequent emergency department visits (**A**) and hospital admissions (**B**). Demographics: age, sex, race and insurance; clinical: depressive symptoms, Systemic Lupus Activity Questionnaire score and Brief Index of Lupus Damage score. CGA, comprehensive geriatric assessment; ED, emergency department; IRR, incidence rate ratio.

Compared with having 0 or 1 CGA-identified conditions, having 2–3, 4–5 and 6 or more CGA-identified conditions was associated with 1.6-fold, 2.1-fold and 2.7-fold higher rates of ED visits; these associations were attenuated and non-statistically significant with adjustment for clinical factors (figure 1A).

### Associations between conditions identified by comprehensive geriatric assessment and hospital admissions

#### Physical performance

Impaired physical performance was not associated with subsequent hospital admissions, regardless of adjustment (table 4). Results were similarly non-statistically significant in sensitivity analyses (online supplemental etable 3).

**Table 4:** Association of conditions identified by CGA with subsequent self-reported hospital admissions

CGA-identified condition (primary definition)	*n*/*N* (%) with any hospital admission	Incidence of admissions (ppy)	Incidence rate ratio (95% CI)*		
			Unadjusted	Adjusted for demographics	Adjusted for demographics and clinical factors
Physical performance					
Impaired	38/136 (27.9)	0.53	1.43 (0.79 to 2.56)	1.41 (0.77 to 2.61)	0.90 (0.46 to 1.76)
Not impaired	22/105 (21.0)	0.37	1.00 (Ref.)	1.00 (Ref.)	1.00 (Ref.)
Cognitive performance					
TMTB or CLOX					
Impaired	54/196 (27.6)	0.53	2.96 (1.20 to 7.26)	2.83 (1.12 to 7.15)	2.22 (0.87 to 5.70)
Not impaired	6/45 (13.3)	0.18	1.00 (Ref.)	1.00 (Ref.)	1.00 (Ref.)
TMTB only					
Impaired	48/153 (31.4)	0.61	3.18 (1.64 to 6.16)	2.84 (1.43 to 5.62)	2.44 (1.20 to 4.96)
Not impaired	12/88 (13.6)	0.19	1.00 (Ref.)	1.00 (Ref.)	1.00 (Ref.)
CLOX					
Impaired	38/132 (28.8)	0.59	1.95 (1.09 to 3.51)	1.65 (0.90 to 3.04)	1.76 (0.93 to 3.32)
Not impaired	22/109 (20.2)	0.30	1.00 (Ref.)	1.00 (Ref.)	1.00 (Ref.)
Self-reported physical function					
Limited	5/32 (15.6)	0.41	0.87 (0.36 to 2.06)	0.79 (0.32 to 1.92)	0.50 (0.16 to 1.58)
Not limited	55/209 (26.3)	0.47	1.00 (Ref.)	1.00 (Ref.)	1.00 (Ref.)
IADLs					
Limited	33/135 (24.4)	0.47	1.03 (0.58 to 1.84)	1.04 (0.57 to 1.90)	0.90 (0.45 to 1.80)
Not limited	27/106 (25.5)	0.45	1.00 (Ref.)	1.00 (Ref.)	1.00 (Ref.)
BADLs					
Limited	21/101 (20.8)	0.51	0.75 (0.42 to 1.35)	0.65 (0.35 to 1.21)	0.47 (0.23 to 0.96)
Not limited	39/140 (27.9)	0.39	1.00 (Ref.)	1.00 (Ref.)	1.00 (Ref.)
Falls					
Yes	20/71 (28.2)	0.62	1.57 (0.86 to 2.88)	1.61 (0.86 to 3.00)	1.75 (0.88 to 3.50)
No	40/170 (23.5)	0.39	1.00 (Ref.)	1.00 (Ref.)	1.00 (Ref.)
Community mobility					
Restricted	27/116 (23.3)	0.47	1.02 (0.57 to 1.82)	0.94 (0.52 to 1.72)	0.74 (0.40 to 1.39)
Not restricted	33/125 (26.4)	0.46	1.00 (Ref.)	1.00 (Ref.)	1.00 (Ref.)
Polypharmacy					
Yes	36/131 (27.5)	0.52	1.33 (0.74 to 2.37)	1.56 (0.84 to 2.87)	1.05 (0.53 to 2.05)
No	24/110 (21.8)	0.40	1.00 (Ref.)	1.00 (Ref.)	1.00 (Ref.)
Urinary incontinence					
Yes	19/78 (24.4)	0.35	0.67 (0.36 to 1.27)	0.75 (0.39 to 1.44)	0.69 (0.34 to 1.39)
No	41/163 (25.2)	0.52	1.00 (Ref.)	1.00 (Ref.)	1.00 (Ref.)

Demographics: age, sex, race and insurance; clinical: depressive symptoms, Systemic Lupus Activity Questionnaire score and Brief Index of Lupus Damage score.

* From negative binomial regression.

BADL, basic activity of daily living; CGA, comprehensive geriatric assessment; CLOX, clock draw; IADL, instrumental activity of daily living; ppy, per person-year; TMTB, Trail Making Test B.

#### Cognitive performance

Impaired cognitive performance was associated with a 3.0-fold higher rate of subsequent hospital admissions; after adjustment for clinical factors, impaired cognitive performance remained associated with a 2.2-fold higher rate of subsequent hospitalisations (table 4). Results were similar in sensitivity analyses (online supplemental etable 3). Impaired TMTB performance was associated with a 3.2-fold higher rate of hospital admissions (2.4-fold higher rate after adjustment for clinical factors), while impaired CLOX performance was associated with a 2.0-fold higher rate of hospital admissions (not statistically significant after adjustment for clinical factors; table 3). Results for TMTB and CLOX impairment were similar in sensitivity analyses, and each additional minute taken in the TMT was associated with a 2.1-fold higher rate of hospital admissions (online supplemental etable 3).

#### Self-reported physical function

Limited self-reported physical function was not associated with subsequent hospital admissions, regardless of adjustment (table 4). Results were similar in sensitivity analyses, and continuous physical function T-scores were similarly not associated with hospital admissions (online supplemental etable 3).

#### Activities of daily living

Neither IADL nor BADL limitations were associated with subsequent hospital admissions, regardless of adjustment (table 4). Sensitivity analyses were similar: neither IADL nor BADL limitations were associated with hospital admissions (online supplemental etable 3).

#### Falls

Self-reported falls in the prior year were associated with higher rates of subsequent hospital visits, but the associations were not statistically significant, regardless of adjustment (table 4). Sensitivity analyses were similar, except that the more restrictive definition of falls was associated with a 2.4-fold higher rate of hospital admissions, and each additional fall was associated with a 21% higher rate of subsequent hospital admissions (online supplemental etable 3).

#### Community mobility

Neither restricted community mobility, regardless of adjustment, nor the continuous UAB-LSA score was associated with hospital admissions (table 4; online supplemental etable 3). The more restrictive definition was associated with a 46% higher rate of hospital admissions, but the association was not statistically significant (online supplemental etable 3).

#### Polypharmacy

Polypharmacy was not statistically significantly associated with subsequent hospital admissions, regardless of adjustment (table 4). Sensitivity analyses showed similar results, but each additional medication was associated with a 7% higher rate of hospital admissions (online supplemental etable 3).

#### Urinary incontinence

Urinary incontinence was not associated with subsequent hospital admissions, regardless of adjustment (table 4). Sensitivity analyses showed similarly non-statistically significant results (online supplemental etable 3).

#### Total number of CGA-identified conditions

The number of CGA-identified conditions was not associated with hospital admissions (figure 1B).

## Discussion

In this population-based study of adults with SLE, we found that conditions identified by several potential components of a CGA, including physical and cognitive performance, self-reported physical functioning, BADLs and falls, were associated with higher rates of subsequent ED visits. Additionally, higher total numbers of these CGA-identified conditions were associated with higher rates of subsequent ED visits. However, most of these associations were no longer statistically significant after adjustment for clinical factors, and community mobility restrictions, polypharmacy and urinary incontinence were not associated with either ED visits or hospital admissions. Further, regardless of adjustment, only cognitive impairment (specifically, TMTB impairment) was associated with a higher rate of subsequent hospitalisation in our study.

Cognitive impairment could increase the risk of ED visits and hospital admissions through multiple mechanisms. Individuals with SLE and cognitive impairment may struggle with SLE treatment management, with lapses leading to the need for acute care. Such individuals may also experience delays in recognising the early signs and symptoms of SLE flares or the development or exacerbation of comorbid conditions, which could necessitate acute care at presentation. Independent of SLE, cognitive impairment could increase the risk of avoidable accidents that require acute care.^40^ Cognitive impairment could also serve as a proxy for aspects of SLE activity and cumulative burden that cannot be directly measured. In our study, the combination of TMTB and CLOX—both of which can be administered quickly and without special equipment in a clinical setting—showed that cognitive impairment was associated with higher rates of subsequent ED visits and hospital admissions, regardless of adjustment. However, individually, only the associations of TMTB impairment with acute care utilisation were statistically significant. This difference may be explained by power (with smaller numbers of individuals being categorised as impaired using the CLOX vs TMTB measure), or by differences in what these tasks measure: the TMTB is used to assess impairment of executive function related to visual attention and task switching,^22^ whereas the CLOX is used to assess various aspects of executive functioning and posterior cortical impairment^26^ and is often used as a screening for dementia^41^ and potential driving issues^42^ among older adults.

We also found that physical performance impairment and self-reported physical functioning limitations were both associated with subsequent ED visits but not hospital admissions. However, the associations of physical performance impairment and self-reported physical functioning limitations with ED visits were at least partially explained by clinical characteristics. We saw similar patterns for IADLs, BADLs and falls, as well as higher total numbers of CGA-identified conditions. Community mobility, polypharmacy and urinary incontinence were not associated with either ED visits or hospital admissions in our study, regardless of adjustment. These results contrast with those seen in older and middle-aged adults, with and without chronic diseases, among whom physical performance impairment,^43^ physical functioning limitations,^44 45^ IADLs and BADLs,^46^ falls,^47^ community mobility^48^ and polypharmacy^49^ have all been associated with higher acute care healthcare utilisation. Although differences in study designs (eg*,* cohort studies,^43^,^4648 49^ meta-analysis,^47^ methods (eg*,* longer follow-up^46 48^; association^4345^,^49^ vs prediction^44^ analyses), populations (eg*,* adults with stroke^44^ and heart failure,^48^ and settings (eg*,* outpatient geriatric clinic,^43^ single health system^45^ and national claims)^49^ likely contribute to these differences, our results suggest that these CGA-identified conditions may not be independently associated with acute care utilisation in the setting of SLE.

Overall, our results suggest that, except for cognitive impairment, CGA-identified conditions may not be useful in predicting acute care utilisation among individuals with SLE, beyond the information on disease activity and damage that rheumatology clinicians already assess. Particularly, our measure of disease damage^38^ includes SLE manifestations that are often quite severe and may mask the effect of CGA-identified conditions. However, future studies addressing these limitations of our study are needed to confirm these results. For example, given that cognitive impairment was assessed with multiple instruments in our study and it was the only factor independently associated with both ED visits and hospitalisation, more comprehensive assessments of the other CGA components may show different results. Our sensitivity analyses also suggest that more restrictive cut-offs for some CGA components (eg*,* IADLs, BADLs and falls) may be useful in predicting subsequent acute care utilisation in this population. Results may also differ with objective outcome assessment (via medical records or claims), since patients’ perceptions and memory can affect the reporting of acute care.

It is important to note that, even if further study corroborates that CGA-identified conditions are not useful in predicting acute care utilisation in the setting of SLE, such evidence would not necessarily negate the value of a CGA in SLE. Identifying these conditions can provide the clinician with a more holistic view of patients and help clinicians make informed shared decisions regarding SLE treatment: for example, simpler regimens could be considered for patients with cognitive impairment, or treatments that do not require frequent visits, could be considered for those with limited community mobility. CGA, with its focus on patient priorities, may also improve the quality of life^50^ and, ultimately, strengthen the patient–clinician bond, particularly if the rheumatology clinician addresses such issues, either directly or through referrals to other specialists who can provide therapy or supportive care.

Other limitations to our study are worth mentioning. There is the potential for differential selection bias, if those who had more ED visits or hospital admissions close to the APPEAL visit (and were thus more likely to have subsequent acute care utilisation), or in the 12 months following the APPEAL visit, were less likely to be included and/or respond to surveys; this would likely result in a bias toward the null. Acute care utilisation was self-reported in this study, which may result in outcome misclassification; this misclassification may be differentially related to the reasons for utilisation or intervening precipitating events, which were also not captured in this study. Additionally, the gap between APPEAL assessments and the assessment of acute care (up to 12 months) could have introduced misclassification of the outcome: unobserved ED visits and hospital admissions in the gap between APPEAL visit and GOAL assessment of outcomes would likely result in non-differential misclassification and a bias toward the null, but misclassification that is differential by exposure cannot be ruled out. As noted above, misclassification of the outcome or other CGA components, including those that were not measured, such as frailty and multimorbidity, could also be affected by cognitive impairment. As with any observational study, there is a possibility of residual confounding by unmeasured or time-varying factors, particularly comorbid conditions, which are incompletely captured by our disease damage measure. Finally, assessments of CGA components in a single research setting may not replicate those in other research settings or in a clinical setting, in which a CGA would likely include probing of the patients, which could help address potential misclassification in surveys (on which participants may either underestimate or overestimate their abilities and activities).

Our study provides initial evidence that identification of some of the components of a CGA, particularly cognitive impairment, may be useful for addressing these issues and mitigating risk of subsequent acute care utilisation in the clinical setting for patients with SLE. Future studies should examine the effectiveness of incorporating CGA measures and geriatrician-led CGA in SLE care, not only in preventing acute care but also in improving other important outcomes, such as patient quality of life and patient satisfaction with clinician encounters.

## Supplementary material

10.1136/lupus-2025-001935 online supplemental file 1

## Data Availability

Data are available upon reasonable request.
