# Supplementary material for: Conditions identified by common comprehensive geriatric assessment measures and subsequent acute care among adults with SLE
Source: Lupus Sci Med. 2026 Apr 8;13(1):e001935. doi: 10.1136/lupus-2025-001935 (PMC13064129; doi:10.1136/lupus-2025-001935)

Supplement: Plantinga *et al*., Conditions Identified by Common Comprehensive Geriatric Assessment Measures and Subsequent Acute Care Among Adults with Systemic Lupus Erythematosus

Table of Contents

eTable 1. Characteristics of participants who were included vs. excluded from this analysis....... 2

eTable 2. Sensitivity analyses: Association of conditions often identified by comprehensive geriatric assessment with subsequent self-reported emergency department visits. 3

eTable 3. Sensitivity analyses: Association of conditions often identified by comprehensive geriatric assessment with subsequent self-reported hospital admissions...................................... 4

eFigure 1. Timing of APPEAL visits (when geriatric conditions were assessed) and the first post-APPEAL GOAL visits with self-reported acute care utilization data. 5

eFigure 2. Inclusion and exclusion of participants for this study. 6

eFigure 3. Conceptual model of associations between geriatric assessment components, acute care utilization, and other participant characteristics... 7

eFigure 4. Distribution of the number of conditions reported by participants that are often identified by comprehensive geriatric assessment screening.. 8

eTable 1. Characteristics of participants who were included vs. excluded from this analysis.

	Value		
**Characteristic**	**Included (*N*=241)**	**Excluded (*N*=210)**	***P*[a]**
Age in years, mean (SD)	46.4 (11.9)	45.9 (11.7)	*0.7*
Age, *n* (%)			*0.5*
18-34 years	53 (22.0%)	39 (18.6%)	
35-49 years	88 (36.5%)	86 (41.0%)	
≥50 years	100 (41.5%)	85 (40.5%)	
Sex at birth, *n* (%)			*0.3*
Female	224 (93.0%)	190 (90.5%)	
Male	17 (7.0%)	20 (9.5%)	
Race, *n* (%)			*0.5*
Black	202 (83.8%)	170 (81.0%)	
White	24 (10.0%)	28 (13.3%)	
Other	15 (6.2%)	12 (5.7%)	
Ethnicity, *n* (%)			*0.7*
Non-Hispanic	226 (94.2%)	199 (94.8%)	
Hispanic	14 (5.8%)	11 (5.2%)	
Education, *n* (%)			*0.9*
High school degree or less	56 (23.2%)	47 (22.4%)	
Some college/associates degree	90 (37.3%)	83 (39.5%)	
College graduate or higher	95 (39.4%)	80 (38.1%)	
Insurance, *n* (%)			*0.9*
None	28 (12.1%)	24 (12.0%)	
Medicaid	44 (19.0%)	38 (19.0%)	
Medicare	84 (36.2%)	79 (39.5%)	
Private	76 (32.8%)	59 (29.5%)	
Clinical			
Disease duration in years, median (IQR)	14.7 (9.2-22.7)	14.8 (9.3-21.2)	*0.5*
SLAQ score, median (IQR)	11 (7-15)	11 (5-16)	*0.9*
BILD score, median (IQR)	2 (1-4)	2 (1-4)	*0.6*
Obese, *n* (%)			*0.4*
Yes	128 (54.7%)	105 (51.0%)	
No	106 (45.3%)	101 (49.0%)	
Currently taking steroid, *n* (%)			*0.4*
Yes	98 (40.8%)	94 (44.8%)	
No	142 (59.2%)	116 (55.2%)	
Currently taking immunosuppressant, *n* (%)			*0.9*
Yes	113 (47.1%)	98 (46.7%)	
No	127 (52.9%)	112 (53.3%)	
Depressive symptoms T-score, mean (SD)	48.5 (8.9)	47.5 (9.4)	*0.3*

[a]By *t*, chi-square, or rank sum test, as appropriate.

eTable 2. Sensitivity analyses: Association of conditions often identified by comprehensive geriatric assessment with subsequent self-reported emergency department visits.

	Incidence rate ratio (95% CI)						
**CGA-identified condition**	**Primary analysis**	**Adjusting for gap[a]**	**Limiting gap[a] to 6 months[b]**	**Using all available data for CGA component[c]**	**Adjusting for SLE medications[d]**	**More restrictive categorization of CGA component**	**CGA component as continuous score**
Physical performance (impaired vs. not)	1.69 (1.17-2.43)	1.71 (1.19-2.46)	1.60 (0.88-2.91)	1.76 (1.24-2.51)	1.67 (1.15-2.40)	1.74 (0.88-3.45)	0.91 (0.84-0.99)
Cognitive performance (impaired vs. not)							
Overall	2.24 (1.32-3.79)	2.26 (1.34-3.82)	2.04 (0.83-5.02)	2.31 (1.37-3.88)	2.21 (1.30-3.74)	1.35 (0.94-1.94)	—
TMTB	1.61 (1.10-2.35)	1.58 (1.08-2.31)	1.20 (0.66-2.17)	1.74 (1.19-2.55)	1.53 (1.05-2.25)	1.68 (1.17-2.40)	1.24 (0.97-1.59)
CLOX	1.30 (0.91-1.87)	1.32 (0.92-1.89)	1.04 (0.58-1.87)	1.45 (1.02-2.06)	1.38 (0.96-1.98)	1.70 (1.13-2.57)	1.00 (0.94-1.06)
Self-reported physical function (limited vs. not)	1.77 (1.10-2.85)	1.83 (1.14-2.95)	2.06 (1.02-4.16)	2.36 (1.55-3.59)	1.73 (1.06-2.82)	1.48 (0.62-3.53)	0.75 (0.61-0.91)
IADLs (limited vs. not)	1.42 (0.99-2.03)	1.47 (1.02-2.12)	1.39 (0.76-2.54)	1.44 (1.03-2.03)	1.37 (0.95-1.96)	1.57 (0.97-2.55)	1.08 (1.02-1.14)
BADLs (limited vs. not)	1.55 (1.09-2.20)	1.55 (1.09-2.21)	1.23 (0.70-2.17)	1.59 (1.15-2.21)	1.50 (1.05-2.13)	1.98 (1.14-3.45)	1.07 (1.06-1.26)
Falls	1.57 (1.08-2.27)	1.57 (1.09-2.28)	1.49 (0.83-2.68)	1.48 (1.02-2.14)	1.62 (1.12-2.34)	1.87 (1.25-2.80)	1.16 (1.04-1.29)
Community mobility (restricted vs. not)	1.04 (0.73-1.49)	1.06 (0.74-1.52)	1.28 (0.73-2.27)	0.97 (0.69-1.37)	1.01 (0.71-1.45)	1.40 (0.96-2.04)	0.98 (0.93-1.03)
Polypharmacy (yes vs. no)	1.24 (0.87-1.78)	1.25 (0.87-1.79)	1.85 (1.05-3.29)	1.38 (0.98-1.96)	1.26 (0.87-1.83)	1.23 (0.80-1.89)	1.05 (0.99-1.12)
Urinary incontinence (yes vs. no)	1.12 (0.77-1.63)	1.12 (0.77-1.63)	1.15 (0.63-2.11)	0.99 (0.68-1.44)	1.06 (0.72-1.57)	1.23 (0.80-1.89)	1.05 (0.99-1.12)

BADLs, basic activities of daily living; CGA, comprehensive geriatric assessment; CLOX, clock draw assessment; IADLs, instrumental activities of daily living; TMTB, Trail Making Test B.

[a]Gap = time between study visit in which CGA-identified problems were assessed and the start of retrospective follow-up for outcomes (maximum of 12 months in primary analysis.

[b]*N*=97.

[c]Physical performance: *N*=270; cognitive performance: *N*=260; TMTB, *N*=261; CLOX, *N*=270; self-reported physical function, *N*=271; IADLs, *N*=266; BADLs, *N*=267; falls, *N*=271; community mobility, *N*=271; polypharmacy, *N*=271; urinary incontinence, *N*=257.

[d]Adjustment for currently taking a steroid and currently taking an immunosuppressant.

eTable 3. Sensitivity analyses: Association of conditions often identified by comprehensive geriatric assessment with subsequent self-reported hospital admissions.

	Incidence rate ratio (95% CI)						
**CGA-identified condition**	**Primary analysis**	**Adjusting for gap[a]**	**Limiting gap[a] to 6 months[b]**	**Using all available data for CGA component[c]**	**Adjusting for SLE medications[d]**	**More restrictive categorization of CGA component**	**CGA component as continuous score**
Physical performance (impaired vs. not)	1.43 (0.79-2.56)	1.57 (0.87-2.85)	5.76 (1.40-23.6)	1.45 (0.86-2.45)	1.41 (0.78-2.54)	1.43 (0.45-4.55)	0.94 (0.83-1.07)
Cognitive performance (impaired vs. not)							
Overall	2.96 (1.20-7.26)	3.10 (1.26-7.60)	5.48 (0.55-54.4)	2.92 (1.26-6.76)	2.93 (1.19-7.21)	2.81 (1.61-4.89)	—[e]
TMTB	3.18 (1.64-6.16)	3.22 (1.66-6.23)	3.98 (1.06-14.9)	3.10 (1.67-5.77)	3.18 (1.64-6.15)	3.20 (1.77-5.80)	2.09 (1.36-3.20)
CLOX	1.95 (1.09-3.51)	2.15 (1.19-3.90)	2.80 (0.83-9.43)	1.95 (1.16-3.31)	1.93 (1.07-3.47)	2.11 (1.06-4.20)	0.93 (0.85-1.02)
Self-reported physical function (limited vs. not)	0.87 (0.36-2.06)	0.92 (0.39-2.20)	0.88 (0.17-4.46)	1.27 (0.63-2.52)	0.90 (0.37-2.20)	—[e]	1.04 (0.76-1.42)
IADLs (limited vs. not)	1.03 (0.58-1.84)	1.16 (0.64-2.10)	2.35 (0.68-8.13)	1.05 (0.62-1.78)	0.98 (0.54-1.78)	0.79 (0.33-1.91)	0.97 (0.87-1.08)
BADLs (limited vs. not)	0.75 (0.42-1.35)	0.75 (0.42-1.35)	0.54 (0.17-1.70)	0.83 (0.49-1.40)	0.75 (0.41-1.35)	0.71 (0.24-2.10)	0.97 (0.83-1.14)
Falls	1.57 (0.86-2.88)	1.63 (0.89-2.98)	2.00 (0.64-6.24)	1.53 (0.90-2.63)	1.54 (0.84-2.83)	2.36 (1.24-4.48)	1.21 (1.00-1.46)
Community mobility (restricted vs. not)	1.02 (0.57-1.82)	1.05 (0.59-1.87)	1.14 (0.37-3.51)	1.03 (0.62-1.71)	1.04 (0.58-1.86)	1.46 (0.79-2.68)	0.95 (0.87-1.03)
Polypharmacy (yes vs. no)	1.33 (0.74-2.37)	1.40 (0.78-2.51)	7.75 (1.96-30.6)	1.37 (0.82-2.30)	1.26 (0.69-2.30)	—[e]	1.07 (0.99-1.15)
Urinary incontinence (yes vs. no)	0.67 (0.36-1.27)	0.65 (0.35-1.23)	0.65 (0.19-2.29)	0.63 (0.35-1.13)	0.68 (0.35-1.31)	0.74 (0.35-1.55)	0.95 (0.84-1.06)

BADLs, basic activities of daily living; CGA, comprehensive geriatric assessment; CLOX, clock draw assessment; IADLs, instrumental activities of daily living; TMTB, Trail Making Test B.

[a]Gap between study visit in which CGA-identified problems were assessed and the start of retrospective follow-up for outcomes (maximum of 12 months in primary analysis).

[b]*N*=97.

[c]Physical performance: *N*=270; cognitive performance: *N*=260; TMTB, *N*=261; CLOX, *N*=270; self-reported physical function, *N*=271; IADLs, *N*=266; BADLs, *N*=267; falls, *N*=271; community mobility, *N*=271; polypharmacy, *N*=271; urinary incontinence, *N*=257.

[d]Adjustment for currently taking a steroid and currently taking an immunosuppressant.

[e]Not estimable.

eFigure 1. Timing of APPEAL visits (when geriatric conditions were assessed) and the first post-APPEAL GOAL visits with self-reported acute care utilization data.

APPEAL, Approaches to Positive, Patient-centered Experiences of Aging with Lupus; GOAL, Georgians Organized Against Lupus.

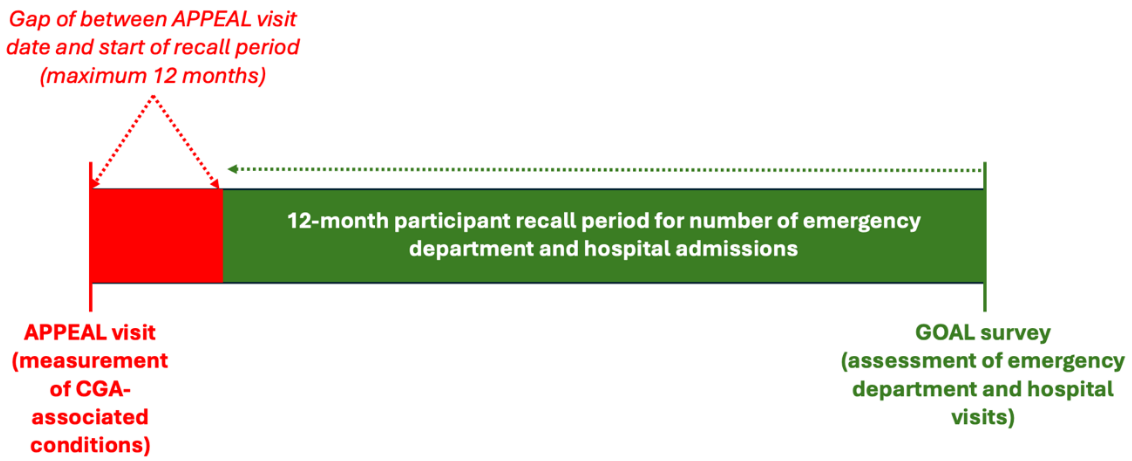

eFigure 2. Inclusion and exclusion of participants for this study.

*APPEAL, Approaches to Positive, Patient-centered Experiences of Aging with Lupus; CGA, comprehensive geriatric assessment. *For Trail Making Test B, clock draw, and urinary incontinence assessments, an additional 23, 4, and 21 observations were missing.*

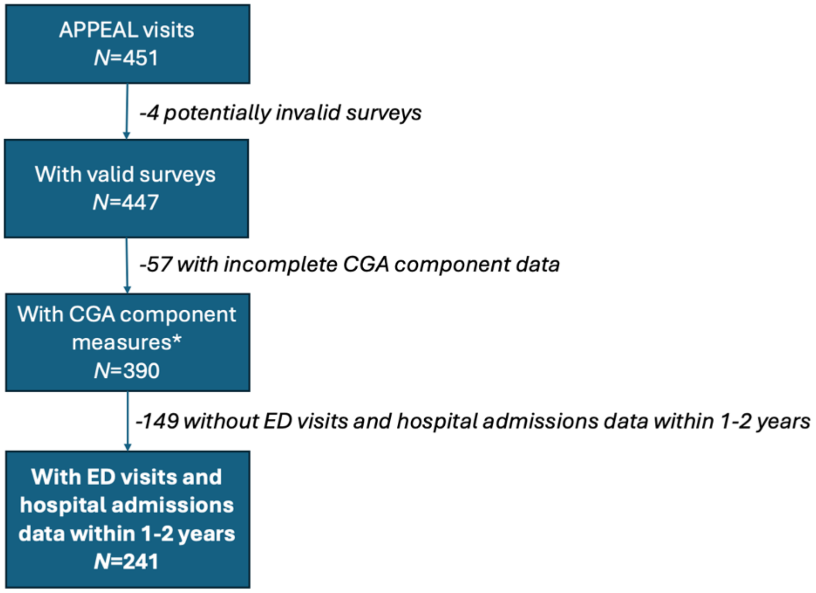

eFigure 3. Conceptual model of associations between geriatric assessment components, acute care utilization, and other participant characteristics.

Green, potential confounder; orange, potential covariate; light blue, potential mediator; gray, unmeasured confounder/mediator. ED, emergency department.

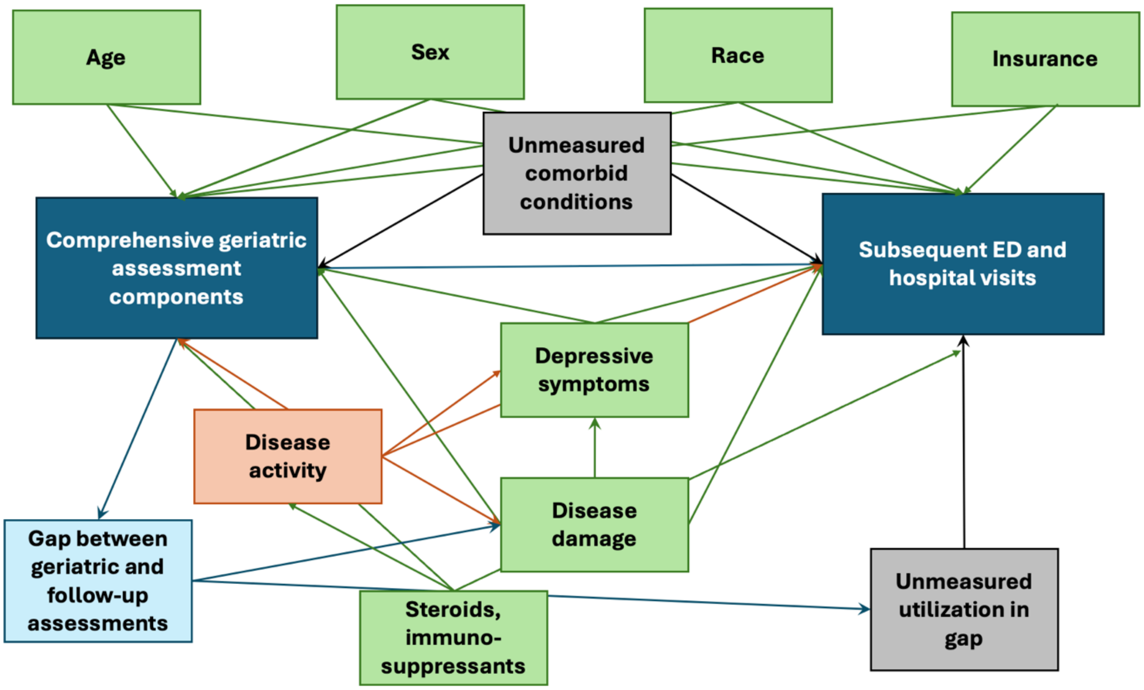

eFigure 4. Distribution of the number of conditions reported by participants that are often identified by comprehensive geriatric assessment screening.

CGA, comprehensive geriatric assessment.

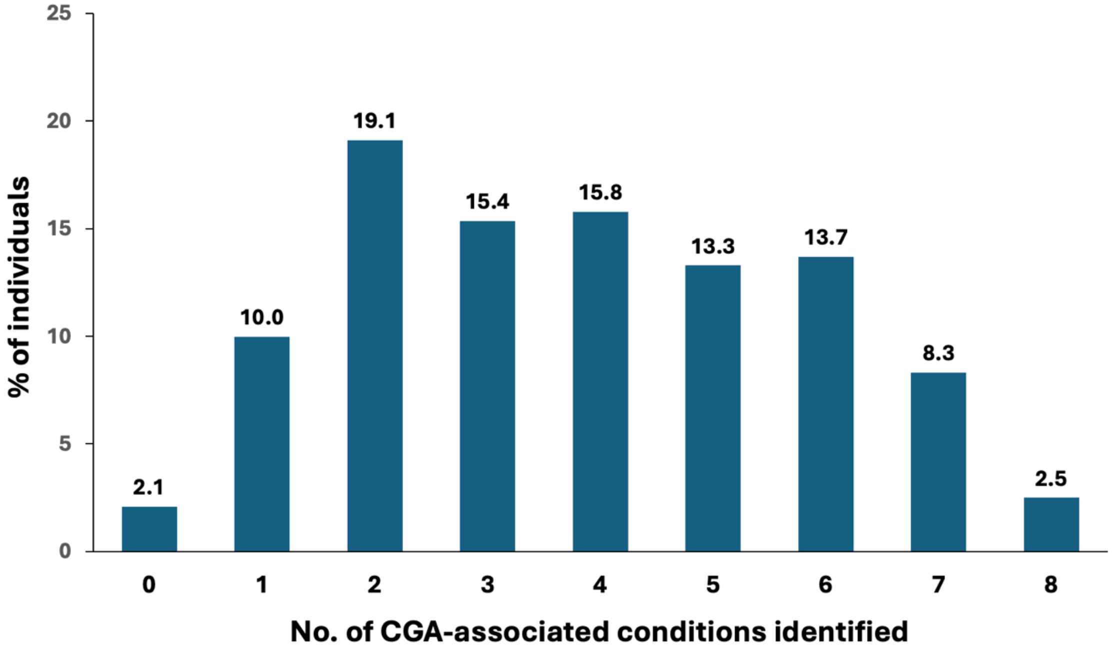

Supplement: online supplemental file 1 [file lupus-13-1-s001.pdf]
